# Supplementary material for: Fitness traits and genetic characterization of a new laboratory colony of Anopheles funestus established in Ifakara, Tanzania
Source: Parasit Vectors. 2026 Aug 13;19:486. doi: 10.1186/s13071-026-07554-3 (PMC13749880; doi:10.1186/s13071-026-07554-3)
Supplement: Supplementary file 1 [file 13071_2026_7554_MOESM1_ESM.docx]

**Supplementary Materials**

**Supplementary Figure 1:** Fluorescent results from the Hydrolysis probe analysis for both FUTAZ and FUMOZ. Quantitative peaks **a)** Clade I; **b)** Clade I and II. Clade I are represented by blue peaks while Clade II indicated by yellow peaks.

**Supplementary Figure 2:** Image of the Gel electrophoresis product after digestion of the D3 region of the Ribosomal DNA; Lane 1 & 29 - Molecular marker (100-bp ladder), Lane 2 & 3 - DNA extraction & PCR negative control respectively, Lane 5-6, 8-10 - M-type, Lane 11-16 - MW-type & Lane 4,7,17-28 - Y-type.
